# Self-assembled multifunctional hairpin probe for ultrasensitive and mismatch-selective miRNA detection

**DOI:** 10.1016/j.jpha.2026.101561

**Published:** 2026-01-22

**Authors:** Baoqiang Chen, Yiping Fan, Qi Wang, Jianguo Xu, Yi Wang, Bingyong Lin, Lee Jia, Longhua Guo

**Affiliations:** a Provincial Key Laboratory of Multimodal Perceiving and Intelligent Systems, Engineering Research Center of Intelligent Human Health Situation Awareness of Zhejiang Province, Jiaxing Key Laboratory of Molecular Recognition and Sensing, College of Biological and Chemical Engineering, Jiaxing University, Jiaxing, Zhejiang, 314001, China; b Key Laboratory of Traditional Chinese Medicine for the Prevention and Treatment of Infectious Diseases in the Elderly in Zhejiang Province (Cultivation), Jiaxing Hospital of Traditional Chinese Medicine, Jiaxing University, Jiaxing, Zhejiang, 314000, China; c School of Biological and Food Engineering, Fuyang Normal University, Fuyang, Anhui, 236037, China; d Department of Oncology, Hefei First People's Hospital, Third Affiliated Hospital of Anhui Medical University, Hefei, 230032, China; e China-Ireland Food & Medicine Innovation Joint Laboratory, The First Affiliated Hospital, Henan University, Kaifeng, Henan, 475004, China

**Keywords:** Multifunctional hairpin probe, Biostability, Palindromic structure, DNA assembly, miRNA analysis, Isothermal amplification

## Abstract

Sensitive, specific, and stable detection of microRNAs (miRNAs) in complex biological environments remains a formidable challenge in molecular diagnostics. We introduce a novel bidirectional palindromic assembled multifunctional hairpin probe (A-MF-HP)—a molecular tool that integrates target recognition, cascade signal amplification, and fluorescence reporting into a single, compact system. Two palindromic arms drive autonomous, bidirectional self-assembly into a nuclease-resistant architecture, enabling robust operation in serum-rich environments. Upon recognition of miRNA-21, a structural switch in one hairpin triggers polymerase extension, nicking, and strand displacement all without auxiliary probes. This initiates a self-propagating disassembly cascade, unfolding additional hairpins and exponentially amplifying the signal. Under the optimized conditions, the platform demonstrates femtomolar-level sensitivity (about 1× 10^−15^ M) across a six-order dynamic range, with single-nucleotide mismatch discrimination and negligible cross-reactivity to unrelated miRNAs. Notably, A-MF-HP retains full functionality after 12 h in 10% human serum, and clinical application to blood samples from lung cancer patients revealed marked fluorescence elevation compared to healthy controls. By uniting biostability, multifunctionality, and autonomous amplification in a single programmable probe, this novel strategy addresses limitations commonly observed in multi-component isothermal amplification assays, such as poor nuclease tolerance and dependence on multiple separate probes, offering a powerful diagnostic tool for miRNA profiling in biomedical and clinical settings.

## Highlights

• A palindromic hairpin probe self-assembles into a nuclease-resistant DNA nanostructure.
• The probe enables target recognition, signal amplification, and reporting in a single system.
• Cascade amplification achieves femtomolar-level sensitivity and discriminates single-base mismatches.
• The system performs reliably in clinical lung cancer samples, confirming diagnostic potential.

## Graphical abstract

**Figure:**
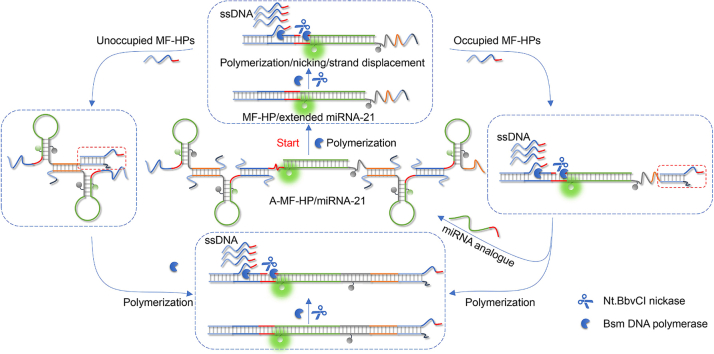

## 1 Introduction

Since the pioneering development of molecular beacons, nucleic acid probes have become indispensable tools in molecular diagnostics [[1], [2], [3]]. Over the past decades, various probe architectures, such as hairpin and allosteric probe designs, have been engineered and integrated with enzymatic components such as polymerases, endonucleases, and ligases to enable powerful signal amplification strategies [[4], [5], [6], [7]]. These systems have demonstrated exceptional sensitivity in detecting disease biomarkers, particularly circulating microRNAs (miRNAs) in blood, which are key indicators in early-stage cancer and other diseases [[8], [9], [10], [11]]. Compared to traditional detection methods, nucleic acid probes offer distinct advantages: they are easy to synthesize, highly consistent across batches, and capable of detecting low-abundance targets with outstanding sensitivity and specificity [[12], [13], [14]]. Their programmability further enhances their diagnostic accuracy, even in complex biological matrices. In particular, miRNA assays have been developed using diverse strategies, including colorimetric approaches, fluorescence-based reporting, and electrochemical sensors [15,16], to achieve ultrasensitive detection. Consequently, they have found wide-ranging applications in disease diagnosis including early cancer screening, infectious disease monitoring, and pathogen detection [17,18].

Despite their advantages, the practical application of nucleic acid probes remains limited by two major challenges. First is the complexity of prevailing probe architectures. Most amplification strategies that combine polymerase extension, site-specific nicking, and strand displacement still rely on multiple, separately designed components such as capture probes, auxiliary templates, and reporter strands. This increases experimental complexity, cost, and the risk of unintended cross-hybridization [[19], [20], [21]]. Each element must be carefully optimized to avoid spurious interactions, yet imperfect control often results in nonspecific amplification and false signals that compromise reliability. Second is the poor biostability of conventional probes [22]. Unmodified nucleic acids are rapidly degraded by endogenous nucleases, especially in serum or other complex biological matrices during long incubations [[23], [24], [25]]. Degradation shortens probe lifetime and produces fragments that raise background fluorescence and reduce accuracy. Chemical modifications can improve nuclease resistance but often weaken hybridization or introduce cytotoxicity, creating new barriers to clinical use. Consequently, current systems either achieve high amplification efficiency at the cost of structural fragility or enhance stability by adding bulky carriers or chemical shields, yet seldom combine both features in one simple probe. Achieving multifunctionality and robust stability within a single, self-sufficient probe remains a critical unmet need [26]. DNA nanoarchitectures with nuclease-resistant features offer promising stability but often sacrifice functional versatility [27,28]. In our previous work, we explored modular multifunctional probes that reduced system complexity, but they also offered limited improvements in biostability [26,29]. Inspired by the unique reverse self-complementary nature of palindromic sequences [30], which have been successfully used in DNA self-assembly for biomedical applications [31,32], we hypothesized that incorporating such motifs into multifunctional probes could enable the formation of spontaneously assembled nanostructures with enhanced biostability to resist nuclease.

Here, we report a multifunctional hairpin probe (MF-HP) incorporating palindromic sequences that enable self-assembly into a nuclease-resistant architecture, assembled MF-HP (A-MF-HP). This design unifies precise target recognition, cascade amplification, and efficient reporting within a single structure through target-induced polymerase replication, site-specific nicking, and strand displacement. Although polymerase extension, nicking, and strand displacement have been widely applied in earlier amplification systems, these methods often rely on multiple probes or auxiliary components, which limits their robustness in complex biological matrices. By combining functional integration with structural stabilization, A-MF-HP provides a simplified yet powerful cascade amplification platform for ultrasensitive miRNA detection, a key oncogenic biomarker [33,34], particularly in complex biological matrices such as human serum. The MF-HP integrates three essential functions within a single hairpin structure: precise target recognition, efficient signal amplification, and robust signal reporting. The palindromic tails enable the probe to adopt a thermodynamically stable conformation through self-assembly, providing resistance to nuclease degradation and ensuring signal transduction efficiency.

## 2 Experimental

### 2.1 Materials and reagents

All oligonucleotides of DNA and miRNAs (Table S1) were synthesized by Sangon Biotech Co., Ltd. (Shanghai, China) and purified by high-performance liquid chromatography (HPLC). A 1× TE buffer (10 mM Tris-HCl, 1 mM ethylenediaminetetraacetic acid (EDTA); pH 8.0 at 25 °C) was used to dissolve all DNA and miRNA sequences to prepare their stock solutions. The 10× CutSmart buffer (200 mM Tris-acetate, 100 mM magnesium acetate, 500 mM potassium acetate, 1 mg/mL bovine serum albumin (BSA), pH 7.9), and Nt.BbvCI endonuclease (10 U/μL) were obtained from New England Biolabs Co., Ltd. (Beijing, China). The 10× Bsm buffer (200 mM Tris-HCl, 100 mM KCl, 100 mM (NH_4_)_2_SO_4_, 20 mM MgSO_4_, 1% (*v/v*) Tween 20, pH 8.8 at 25 °C), and Bsm DNA Polymerase (Large Fragment, 8 U/μL) were supplied by Thermo Scientific Co., Ltd. (Shanghai, China). Deoxynucleotide triphosphates (dNTPs; 25 mM), NTP set (100 mM each), 1× TBE electrophoresis buffer (90 mM Tris, 90 mM boric acid, 10 mM EDTA, 7 M urea, pH 8.0), a 29:1 acrylamide-bisacrylamide solution (30%), ammonium persulfate (APS), 10000× 4S Red Plus, and 6× DNA loading buffer were obtained from Sangon Biotech (Shanghai) Co., Ltd. (Shanghai, China). Ultrapure water with a resistance of 18.2 MΩ, generated using a Milli-Q Water Purification System (Millipore, Burlington, MA, USA), was employed in this study.

### 2.2 Instruments

Fluorescence measurements were performed according to our previously reported method with minor modifications [35]. The measurements were performed using a Spark Multimode Microplate Reader (Tecan, Männedorf, Switzerland) with the following parameters: excitation wavelength of 460 nm, excitation bandwidth of 20 nm, emission wavelength step size of 1 nm, 10 flashes, recording range from 505 to 650 nm, and a manual gain setting of 80. Electrophoresis analysis was conducted with a Servicebio PW-600 electrophoresis analyzer (Servicebio, Wuhan, China), and gel images were captured using a ChampGel 7000 gel imaging system (SageCreation, Beijing, China). The reaction temperature was consistently controlled using a Bio-Rad T100 thermal cycler system (Hercules, CA, USA).

### 2.3 Preparation of A-MF-HP

To construct A-MF-HP, the MF-HP was used by dissolving it in 1× TE buffer containing 15 mM MgCl_2_. The MF-HP was then kept at 95 °C for 5 min and then allowed to cool naturally to room temperature for 0.5 h to self-assembly into A-MF-HP. Prior to usage, the A-MF-HP was stored at 4 °C.

### 2.4 A-MF-HP-based target miRNA detection

To analyze miRNA-21, a reaction mixture was prepared by incubating 3.5 μL of double distilled water (dd-H_2_O), 2 μL of A-MF-HP, 0.5 μL of target miRNA-21 at a specific concentration, 0.5 μL of Bsm DNA polymerase (8 U/μL), 1 μL of 10× Bsm buffer, 0.5 μL of Nt.BbvCI (10 U/μL), 1 μL of 10× CutSmart buffer, and 1 μL of 25 mM dNTPs at 37 °C for 60 min. This combined buffer was chosen because Bsm DNA polymerase and Nt.BbvCI nicking endonuclease each require different optimal buffers, and no single buffer could fully support both enzymatic activities. The resultant solution was measured with a final pH value of 7.75. After the incubation, 190 μL of dd-H_2_O was added, and the samples were immediately transferred to a 96-well fluorescence microplate (Servicebio, Wuhan, China) for fluorescence measurement. It is important to note that the final concentrations of all oligonucleotides were calculated based on the total volume of the final 200 μL reaction mixture. The assay performance was evaluated by measuring the fluorescence intensity at 521 nm in both the presence and absence of the target miRNA. For real sample analysis, the human blood samples were collected by Fuyang People's Hospital and approved by the Ethics Committee of Clinical Trials (Approval No.: 2023byzd219). Written informed consent was obtained prior to sample collection. The human blood samples were collected using vacuum tubes containing a coagulation activator. The samples were allowed to stand at room temperature for 20–30 min to ensure complete coagulation, followed by centrifugation at 5000 rpm for 10 min to separate the serum. An aliquot of 400 μL of the supernatant serum was transferred into sterile cryogenic tubes, labeled, and stored at −80 °C until use.

For real sample analysis, total RNA was isolated from human serum using a commercial serum miRNA extraction kit (HaiGene Biotech Co., Ltd., Harbin, China) following the manufacturer's protocol and previously reported methods [36]. Briefly, 300 μL of serum was thoroughly mixed with 800 μL of miRNA reagent and incubated at room temperature for 5 min. The mixture was centrifuged at 13,000 rpm for 5 min, and the supernatant was collected and combined with 1 mL of isopropanol. The resulting solution was loaded onto the miRNA adsorption column in three portions, each followed by centrifugation at 13,000 rpm for 15 s. The column was subsequently washed with 700 μL of 75% isopropanol and 500 μL of absolute ethanol under the same centrifugation conditions. After a final 2 min centrifugation at 13,000 rpm and a brief air-drying step at room temperature to remove residual ethanol, bound miRNAs were eluted with RNase-free water. The concentration and purity of the extracted miRNAs were determined spectrophotometrically at 260 nm using a NanoDrop™ instrument (Thermo Fisher Scientific, Vantaa, Finland). The purified total miRNAs were subsequently analyzed using our described assay or converted into complementary DNA (cDNA) for reverse transcription (RT)-quantitative polymerase chain reaction (qPCR) analysis.

### 2.5 Polyacrylamide gel electrophoresis (PAGE) analysis

A 12% native-PAGE gel was prepared according to our previously reported method with minor modifications [37], by mixing 7.9 mL of dd-H_2_O, 8 mL of 30% acrylamide: bis-acrylamide solution, 4 mL of 10× TBE buffer, 160 μL of 10% ammonium persulfate, and 15 μL of N,N,N′,N′-tetramethylethylenediamine (TEMED) (Servicebio, Wuhan, China). The gel was left at room temperature for approximately 30 min to polymerize. For electrophoresis, each sample well was loaded with a mixture containing 10 μL of the test sample, pre-mixed with 3 μL of 6× DNA loading buffer, 3 μL of 100× 4S Red Plus, and 2 μL of dd-H_2_O. Electrophoresis was conducted in 1× TBE buffer at a constant voltage of 130 V for 1 h. The gel image was subsequently captured using a gel imaging system.

### 2.6 RT-qPCR based real sample analysis

RT was carried out in accordance with the RT kit instructions. For genomic DNA (gDNA) removal, 1 μL of 10× gDNA remover buffer, 1 μL of gDNA remover, 2 μL of RNA solution (250 ng/μL), and 6 μL of nuclease-free water were mixed and incubated at 37 °C for 2 min, then chilled on ice. The subsequent 20 μL RT reaction contained 10 μL of treated RNA, 4 μL of 5× reaction buffer, 1 μL of SweScript RT II Enzyme Mix, 1 μL of 100 μM stem-loop RT primer, and 4 μL of nuclease-free water. The temperature program was 25 °C for 5 min, 55 °C for 15 min, and 85 °C for 5 s. The resulting cDNA was stored at −80 °C for later analysis.

RT-qPCR was performed in a 20 μL reaction system containing 10 μL of 2× SYBR Green qPCR Master Mix (No 6-carboxy-X-rhodamine) (Servicebio, Wuhan, China), 1 μL each of 10 μM forward and reverse primers, 2 μL of cDNA, and 6 μL of RNase-free water. The amplification program comprised an initial denaturation at 95 °C for 30 s, followed by 40 cycles of 95 °C for 15 s and 60 °C for 30 s. Relative expression of miR-155 was calculated using the *ΔC*_*q*_ method (*ΔC*_*q*_ = *C*_*q*(miR-21)_-*C*_*q*(U6)_). Each sample was analyzed in triplicate on a Bio-Rad CFX Connect Real-Time PCR System (Bio-Rad Laboratories, Inc., Hercules, CA, USA), with reaction temperatures controlled by a Bio-Rad T100 thermal cycler (Bio-Rad Laboratories, Inc., Hercules, CA, USA). Primer sequences are listed in Table S1.

## 3 Results and discussion

### 3.1 Probe architecture and structural features

**Scheme 1:**
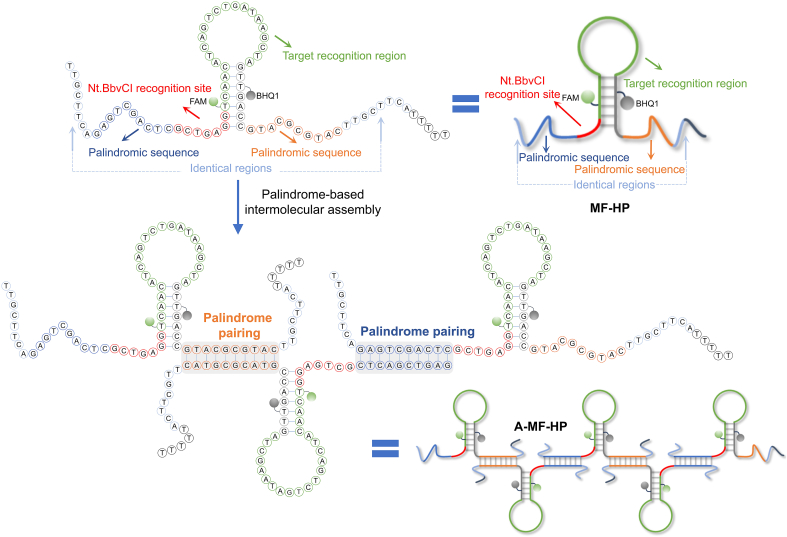
Structure analysis of multifunctional hairpin probe (MF-HP) and bidirectional palindromic assembly of MF-HP into an assembled MF-HP (A-MF-HP). FAM: carboxyfluorescein; BHQ1: black hole quencher.

As shown in Scheme 1, the MF-HP is strategically designed with several key domains: (i) a 21-nt target recognition sequence (5′-TCAACATCAGTCTGATAAGCTA-3′, green bases) in the loop region, (ii) a partial Nt.BbvCI nicking site (5′-GCTGAGG-3′, red bases) adjacent to a 7-bp intramolecular stem (5′-GGTCAAC-3'/5′-GTTGACC-3′), (iii) two distinct palindromic sequences (5′-CTACGCGTAG-3′, blue bases; 5′-GTACGCGTAC-3′, orange bases) enabling nanostructure assembly, and (iv) identical 8-nt sequences (5′-TTGCTTCA-3′, light blue bases) for structural control. The 3′-end incorporates a T5 (5′-TTTTT-3′, black bases) spacer to prevent nonspecific polymerization and a fluorescent resonance energy transfer (FRET) pair (5-carboxyfluorescein (5-FAM)/black hole quencher (BHQ1)) positioned on opposing strands of the stem structure, achieving efficient quenching in the closed conformation. This architecture allows spontaneous formation of A-MF-HP through bidirectional palindromic structure based intermolecular hybridization while maintaining target accessibility in the loop region. The stem-loop configuration provides dual functionality by both suppressing background fluorescence through spatial proximity of the FRET pair (<10 nm) and serving as a structural switch for target detection.

### 3.2 Target detection and signal amplification mechanism

**Scheme 2:**
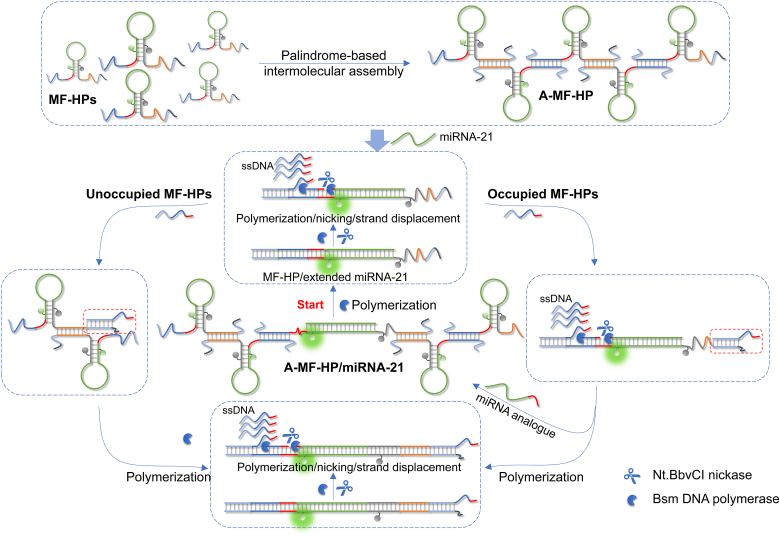
Schematic illustration of an assembled multifunctional hairpin probe (A-MF-HP) based microRNA (miRNA) detection, where miRNA binding triggers MF-HP hairpin opening, initiating polymerase replication, nicking, and strand displacement cycles, leading to fluorescence recovery and cascaded amplification of single-stranded DNA (ssDNA) products within the A-MF-HP nanoarchitecture.

Upon target miRNA recognition (Scheme 2), specific hybridization with the exposed loop region of MF-HP forms an A-MF-HP/miRNA-21 complex and induces a conformational change that destabilizes one MF-HP stem. This transition spatially separates the FAM fluorophore from the BHQ1 quencher, resulting in fluorescence recovery as the primary amplified signal. Concurrently, Bsm DNA polymerase extends the 3′ end of the hybridized miRNA using MF-HP as a template, generating a duplex containing a nicking endonuclease recognition site. The nicking enzyme introduces a single-strand nick to produce a 3′-hydroxyl terminus, which primes further polymerase-mediated extension and displaces downstream single-stranded DNA (ssDNA). Iterative cycles of polymerization, nicking, and strand displacement yield exponential amplification, peeling off MF-HP units and continuously generating ssDNA products. Meanwhile, the increased rigidity of the MF-HP upon duplex formation with extended miRNA-21 contributes to further fluorescence enhancement.

Importantly, the generated ssDNA products can hybridize either with unoccupied MF-HPs remaining on the A-MF-HP scaffold or with already target-bound occupied MF-HPs to further propagate the amplification cascade. In the former case, ssDNA initiates a strand polymerization-nicking-displacement cycle, similar to that triggered by the original miRNA-21 target, thereby producing additional ssDNA copies. In the latter case, the ssDNA binding to target-occupied MF-HPs ssDNA also initiates a strand polymerization-nicking-displacement cycle, the difference is that this process leads to the additional displacement of target miRNA analogues, which contains two extra bases compared to the original miRNA, but maintain full hybridization compatibility. These miRNA analogues can then participate in subsequent amplification cycles by interacting with either unbound or target-bound MF-HPs on A-MF-HP, propagating the reaction. Theoretically, this cascaded amplification process can unfold a substantial number of MF-HP hairpin structures, resulting in a dramatically amplified fluorescence signal. As a result, the system achieves ultrasensitive detection where even trace amounts of target miRNA can trigger a self-sustaining amplification cascade. In contrast, under target-negative conditions, the intrinsic palindromic sequences mediate the spontaneous self-assembly of MF-HPs into highly ordered A-MF-HP nanostructures. This compact configuration not only confers nuclease resistance through steric shielding but also enhances thermodynamic stability via multivalent base pairing. Consequently, the tightly packed architecture leads to effective fluorophore quenching, thereby minimizing background signal leakage.

### 3.3 Feasibility demonstration of the A-MF-HP-based cascade amplification system

**Fig. 1:**
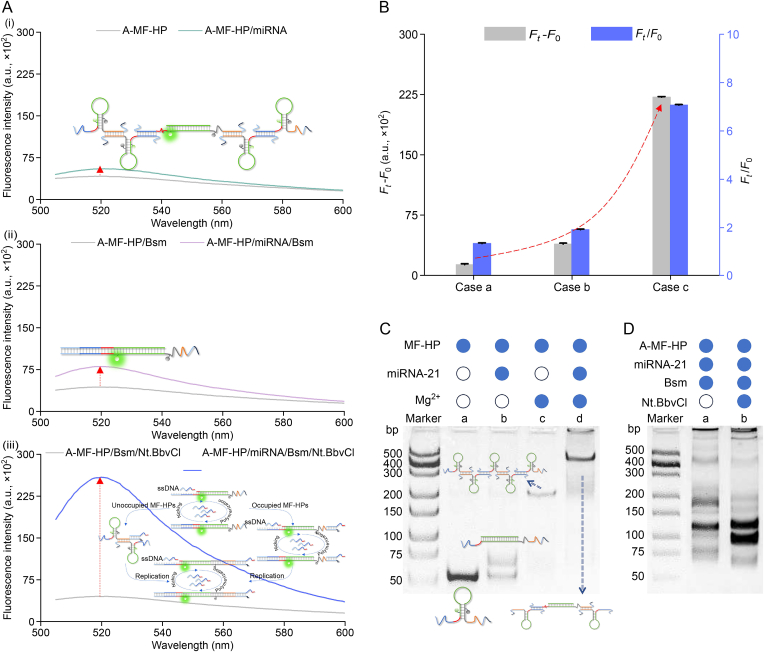
Feasibility demonstration and mechanistic validation of the assembled multifunctional hairpin probe (A-MF-HP)-based cascade amplification system for microRNA-21 (miRNA-21) detection. (A) Fluorescence spectra under different cases: A-MF-HP with/without miRNA-21 (i); A-MF-HP with/without miRNA-21 in the presence of Bsm DNA polymerase (ii); A-MF-HP with/without miRNA-21 in the presence of Bsm polymerase and Nt.BbvCI nickase (iii). (B) Quantitative analysis of fluorescence responses from cases a–c, including net signal gain (*F*_t__*-*_*F*_0_) and signal-to-noise ratio (*F*_t_/*F*_0_). (C) Native-polyacrylamide gel electrophoresis (PAGE) analysis: (a) 400 nM MF-HP alone, (b) duplex formed by hybridization of 400 nM MF-HP with 400 nM miRNA-21, (c) 400 nM MF-HP assembled into A-MF-HP in the presence of 15 mM Mg^2+^, (d) A-MF-HP hybridized with 400 nM miRNA-21 and 15 mM Mg^2+^. (D) PAGE verification of enzymatic reactions: (a) A-MF-HP and miRNA-21 (200 nM) incubated with 4 U Bsm DNA polymerase, (b) A-MF-HP and miRNA-21 (200 nM) treated with both 4 U Bsm DNA polymerase and 5 U Nt.BbvCI nicking endonuclease.

To evaluate the feasibility and operational mechanism of the proposed A-MF-HP-based amplification system, we systematically characterized its performance via fluorescence spectral analysis. As shown in panel a of Fig. 1A, the introduction of target miRNA-21 to the A-MF-HP scaffold resulted in a slight but distinct fluorescence recovery (case a), confirming successful hybridization and initial stem-loop disruption. Upon the addition of Bsm DNA polymerase to the A-MF-HP and A-MF-HP/miRNA-21 complex (panel b), further fluorescence enhancement was observed (case b). This enhancement reflects the enzyme's ability to extend the miRNA using the MF-HP as a template, leading to more effective hairpin unfolding and signal enhancement compared to A-MF-HP alone. Most notably, in the presence of both Bsm DNA polymerase and the Nt.BbvCI nicking enzyme (panel c), a significantly enhanced fluorescence signal was observed. This result indicates that the complete cascaded amplification cycle comprising iterative polymerase replication, site-specific nicking, and strand displacement, significantly improves the signal output beyond that of the previous conditions (case c). Fig. 1B presents the corresponding quantitative analysis, including the net signal gain and signal-to-noise ratio, defined as *F*_t_/*F*_0_ and *F*_t_-*F*_0_, respectively. Here, *F*_t_ and *F*_0_ represent the peak fluorescence intensities with and without the presence of miRNA-21. The observed trend in fluorescence intensity (c > b > a) provides strong evidence supporting the system's specific target recognition, polymerase-mediated amplification, and exponential signal enhancement enabled by the coordinated enzymatic cascade. Reaction kinetics study to demonstrate the dynamic amplification mechanism of the cascade system's reaction is provided in Fig. S1. Collectively, these results validate the design rationale of the A-MF-HP sensing system and highlight its potential for ultrasensitive miRNA detection. The clear correlation between enzymatic components and signal amplification efficiency further reveals the robustness and effectiveness of this cascade amplification strategy.

### 3.4 Gel electrophoresis characterization

To validate the self-assembly of A-MF-HP nanostructure, native PAGE was employed. As shown in lane a of Fig. 1C, MF-HP alone produced a distinct band corresponding to about 50 bp, consistent with its expected molecular size. Upon hybridization with the target miRNA (lane b), a new, slower-migrating band appeared above the original MF-HP band, indicating the formation of an MF-HP/miRNA duplex and confirming specific target recognition and hybridization. Subsequent annealing of MF-HP in the presence of 15 mM Mg^2+^ resulted in the emergence of a high-molecular-weight band in lane c, migrating at a position corresponding to approximately 200 bp. This shift suggests successful palindromic-sequence-guided self-assembly of MF-HP into a higher-order architecture, denoted as A-MF-HP. Based on the migration distance and molecular weight estimation, it is inferred that approximately four MF-HP units assembled into each A-MF-HP structure. Notably, upon the addition of miRNA, an additional, slower-migrating band was observed above the A-MF-HP band in lane d, indicating the formation of an A-MF-HP/miRNA complex and demonstrating that A-MF-HP remains functionally active for target capture post-assembly. On this basis, we further investigated the behavior of the A-MF-HP/miRNA complex in the presence of polymerase and nicking enzyme. As shown in lane a of Fig. 1D, treatment of the A-MF-HP/miRNA complex with Bsm DNA polymerase alone resulted in a noticeable diffusion of the A-MF-HP band, accompanied by the appearance of several lower molecular weight bands. This observation suggests that the polymerase extended the hybridized miRNA using the MF-HP sequence as a template, thereby disrupting the hairpin structure and leading to partial disassembly of the A-MF-HP nanostructure. Notably, a distinct band appeared between the 100 and 150 bp markers, which can be attributed to the formation of MF-HP/extended miRNA duplexes. Other faint bands likely correspond to residual A-MF-HP fragments of different sizes and some weak byproducts. A similar phenomenon was observed in lane b, where the A-MF-HP-miRNA complex was co-incubated with both Bsm DNA polymerase and Nt.BbvCI nicking enzyme. In this case, additional bands were detected below the MF-HP/extended miRNA duplex, which indicate the nicked counterpart generated by site-specific cleavage of the extended duplex as well as duplexes of MF-HP with ssDNA. Because the fluorescent dye preferentially binds to double-stranded DNA, the ssDNA amplicons could not be visualized. These results collectively confirm that the A-MF-HP architecture not only facilitates specific miRNA binding but also serves as a functional template for polymerase-mediated extension and nickase-mediated cleavage. It should be noted that although Bsm polymerase alone or in combination with Nt.BbvCI endonuclease may produce some byproducts [[38], [39], [40]], the fluorescence background signals in panel b and panel c of Fig. 1A remained very close to that of panel a. These results suggest that the assay using Bsm DNA polymerase and Nt.BbvCI endonuclease is essentially free from background signal interference.

### 3.5 Demonstration of ssDNA amplification capacity

**Fig. 2:**
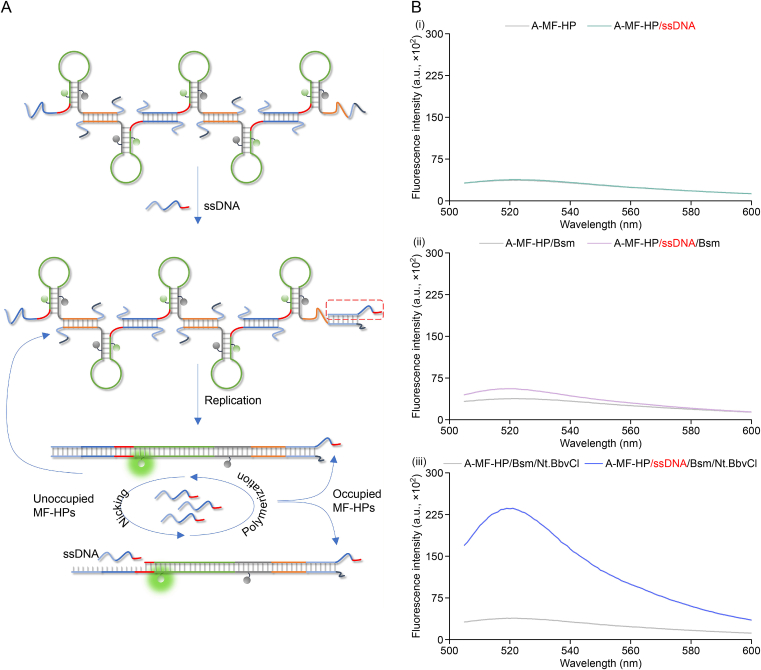
Fluorescence verification of the amplification capability of synthetic single-stranded DNA (ssDNA) products. (A) A schematic diagram depicting the outline of ssDNA-induced signal amplification with an assembled multifunctional hairpin probe (A-MF-HP). (B) Fluorescence verification of ssDNA-mediated cascade amplification: A-MF-HP (20 nM), A-MF-HP (20 nM) + ssDNA (10 nM) (i) ; A-MF-HP (20 nM) + Bsm DNA polymerase (20 U/mL), A-MF-HP (20 nM) + ssDNA (10 nM) + Bsm DNA polymerase (20 U/mL) (ii); A-MF-HP (20 nM) + Bsm DNA polymerase (20 U/mL) + Nt.BbvCI nickase (25 U/mL), A-MF-HP (20 nM) + ssDNA (10 nM) + Bsm DNA polymerase (20 U/mL) + Nt.BbvCI nickase (25 U/mL) (iii). Experimental conditions: deoxynucleotide triphosphates (dNTPs) = 125 μM, *t* = 60 min.

To experimentally validate the critical role of ssDNA products in the cascade amplification process, we designed synthetic ssDNA fragments to evaluate their ability to trigger signal amplification in the A-MF-HP system under different cases. Fig. 2A provides a schematic overview of the signal amplification process mediated by ssDNA. As shown in Fig. 2B, when ssDNA was introduced as a substitute for target miRNA-21 and incubated with A-MF-HP (panel a), either alone or in the presence of Bsm DNA polymerase (panel b) or both Bsm DNA polymerase and Nt.BbvCI nickase (panel c), the resulting fluorescence responses closely resembled those observed during target detection (Fig. 1A), exhibiting comparable emission spectra and similar amplification efficiency. Due to the different binding sites of ssDNA and the target miRNA-21 on the MF-HP, the only distinction is that direct hybridization of ssDNA with MF-HP induces negligible fluorescence response. This result confirms that ssDNA products function as efficient intermediates in the amplification cascade, capable of propagating the reaction through hybridization with MF-HPs or displacement of target analogues from occupied probes.

### 3.6 Quantitative detection of miRNA-21 using the A-MF-HP amplification system

**Fig. 3:**
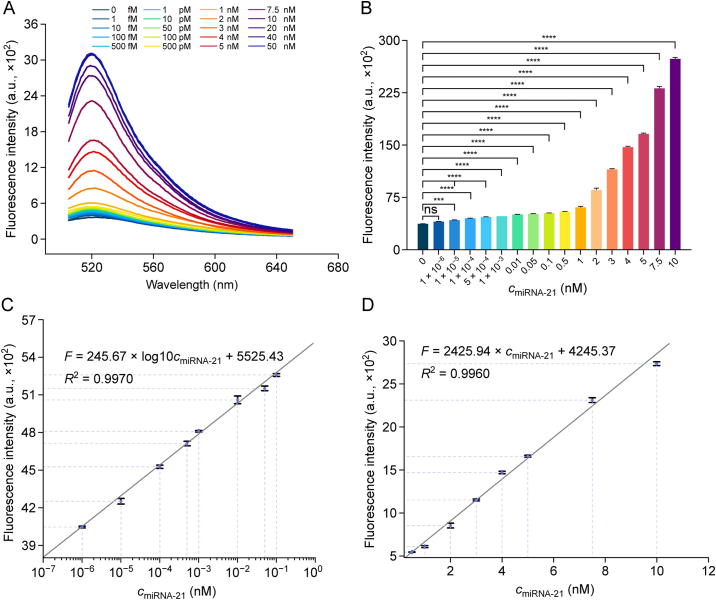
Quantitative detection performance of the assembled multifunctional hairpin probe (A-MF-HP)-based cascade amplification system toward microRNA-21 (miRNA-21). (A) Fluorescence emission spectra of A-MF-HP incubated with increasing concentrations of miRNA-21 (0−50 nM). (B) Peak fluorescence intensities at 521 nm vs. concentration. The coefficient of variation (*CV*) values for miRNA-21 ranging from 1 fM to 10 nM are 0.19%, 0.18%, 0.53%, 0.29%, 0.37%, 0.13%, 0.61%, 0.36%, 0.21%, 0.42%, 1.88%, 3.24%, 0.99%, 0.99%, 0.70%, 1.18% and 0.79%, respectively. *CV*_average_ = 0.77%. (C) Calibration curve showing logarithmic response at low concentrations (1 fM−100 pM). (D) Linear response curve at higher concentrations (500 pM−10 nM). The data represent mean values from three repetitive experiments. Experimental conditions: Bsm = 20 U/mL, Nt.BbvCI = 25 U/mL, deoxynucleotide triphosphates (dNTPs) = 125 μM, MF-HP = 20 nM, t = 60 min. ^∗∗∗^*P* < 0.001, *^∗∗∗∗^P* < 0.0001, ns: not significant.

Reliable quantification of miRNAs is critical for early disease diagnostics and therapeutic monitoring. To demonstrate the sensitivity and dynamic range of our A-MF-HP-based amplification system, we performed fluorescence-based detection of miRNA-21 across concentrations ranging from 1 fM to 40 nM under optimized isothermal conditions (Fig. S2). As shown in Fig. 3A, fluorescence intensity progressively increased with miRNA-21 concentration, indicating successful target binding, signal amplification, and structural transform of the MF-HP probes. The overlap of fluorescence spectra at 40 nM and 50 nM indicates the complete consumption of MF-HP probes on the A-MF-HP scaffold. The corresponding dose-response were illustrated in Fig. 3B. The *CV* values ranged from 0.13% to 3.24%, all within 4%, demonstrating good reproducibility of the assay. In the low concentration range (1 fM-100 pM) (Fig. 3C), the system followed a logarithmic response: *F* = 245.67 × log10 *c*_miRNA-21_ + 5525.43 (*R*^2^ = 0.9970), yielding an estimated limit of detection (LOD) of about 1 fM (approximately 1× 10^−15^ M) (3σ method). At higher concentrations (500 pM–10 nM) (Fig. 3D), fluorescence exhibited a strong linear correlation: *F* = 2425.94 × *c*_miRNA-21_ + 4245.37 (*R*^2^ = 0.9960). This dual-response behavior can be attributed to the concentration-dependent amplification mechanism. At low target concentrations, only a limited number of MF-HP units on the A-MF-HP are activated by miRNA-21, initiating the production of ssDNA intermediates that drive exponential signal amplification. In contrast, at high target concentrations, a majority of the MF-HPs on A-MF-HP are directly hybridized with miRNA-21, leading to rapid signal generation primarily through target binding, with a relatively diminished contribution from ssDNA-mediated amplification. Comparative analysis with existing methods (Table S2) revealed superior performance in sensitivity (LOD), dynamic range, and amplification efficiency. Notably, our method offers exceptional operational simplicity, requiring only sample mixing and incubation, unlike conventional approaches that involve complex nanoparticle synthesis or surface modifications. These findings validate the A-MF-HP platform as a powerful and practical tool for ultrasensitive miRNA analysis, with strong potential for practical diagnostics.

### 3.7 Selectivity of the A-MF-HP system

**Fig. 4:**
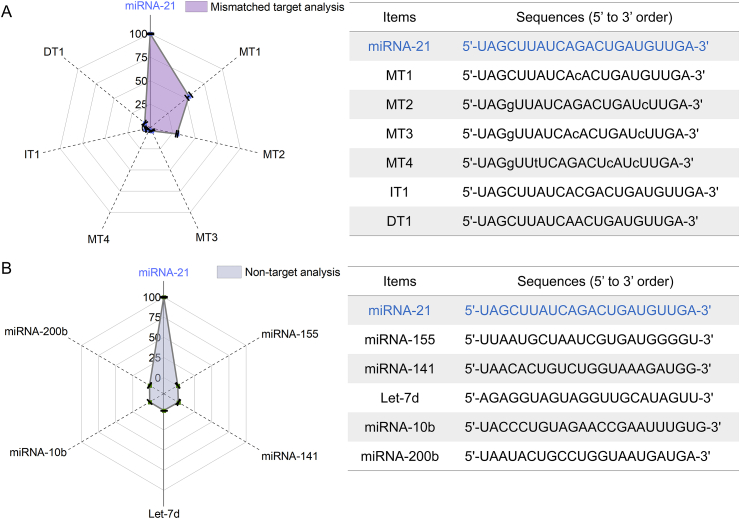
Selectivity evaluation of the assembled multifunctional hairpin probe (A-MF-HP) amplification system. (A) Fluorescence responses of the system to microRNA-21 (miRNA-21) and its sequence variants, including single-base mismatch (MT1), two-base mismatch (MT2), three-base mismatch (MT3), four-base mismatch (MT4), one-base insertion (IT1), and one-base deletion (DT1). (B) Fluorescence responses to non-target miRNAs (miRNA-155, miRNA-141, let-7d, miRNA-10b, and miRNA-200b), showing negligible signals. Experimental conditions: target or variant = 10 nM, Bsm = 20 U/mL, Nt.BbvCI = 25 U/mL, deoxynucleotide triphosphates (dNTPs) = 125 μM, MF-HP = 20 nM, T = 60 min. Error bars are obtained from three repetitive tests.

Previous studies have documented the high sequence homology among miRNAs, making specificity a critical criterion for accurate detection. To assess the selectivity of our A-MF-HP-based amplification system, we tested its ability to discriminate between perfectly matched miRNA-21 and a panel of synthetic variants with increasing degrees of sequence mismatch: single-base mismatch (MT1), two-base mismatch (MT2), three-base mismatch (MT3), four-base mismatch (MT4), as well as one-base insertion (IT1) and one-base deletion (DT1). To clearly evaluate probe selectivity, the relative fluorescence intensity (*RFI*) was calculated using the equation *RFI* = (*F* – *F*_0_)/(*F*_t_ – *F*_0_) × 100%, where *F*, *F*_t_, and *F*_0_ represent the fluorescence intensities obtained from the test sample, the target miRNA-21, and the blank control (without any targets), respectively. As shown in Fig. 4A, although all mismatched sequences elicited some fluorescence enhancement, the signal intensities were markedly lower than that of the perfectly matched target. Specifically, MT1 retained only 53.6% of the fluorescence intensity observed for miRNA-21, while MT2 dropped to 29.8%. Further mismatches led to even weaker signals, with MT3 (3.9%), MT4 (2.5%), IT1 (5.3%), and DT1 (7.6%) all producing less than 10 % of the fluorescence intensity of miRNA-21, indicating a significant loss of hybridization efficiency and amplification capability. To further verify specificity, five unrelated miRNAs (miRNA-155, miRNA-141, let-7d, miRNA-10b, and miRNA-200b) were tested under identical conditions. As depicted in Fig. 4B, these non-target sequences produced minimal fluorescence, all below 2.0% relative to the miRNA-21 response, and closely aligned with the background level. This signal behavior observed in Fig. 4 is consistent with the design of the A-MF-HP system: only perfect or near-perfect base pairing at the loop region of MF-HP effectively triggers conformational switching and subsequent enzymatic amplification. Mismatches disrupt duplex stability and reduce polymerase extension efficiency, thereby impeding signal generation. These findings suggest that the A-MF-HP method offers high selectivity, ensuring minimal cross-reactivity.

### 3.8 Evaluation of biostability in A-MF-HP nanoarchitectures

**Fig. 5:**
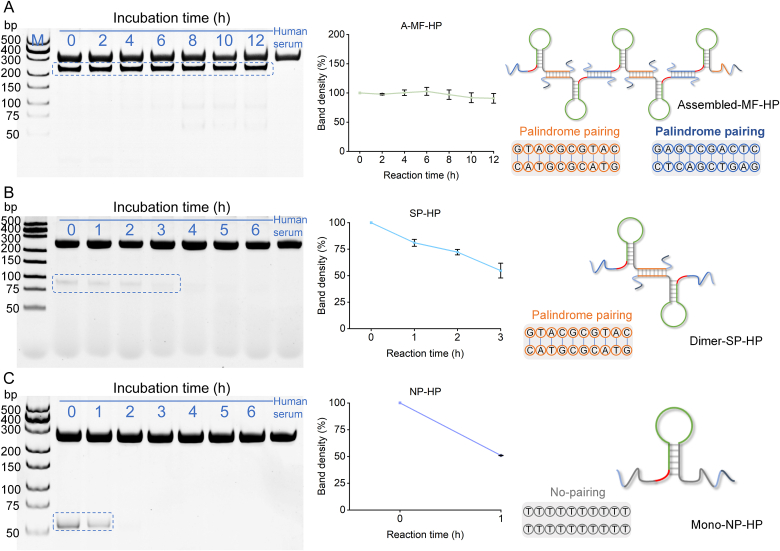
Gel electrophoresis analysis comparing the nuclease stability of different hairpin probe architectures in 10% human serum over defined incubation times. (A) An assembled multifunctional hairpin probe (A-MF-HP). (B) Dimeric single-palindromic hairpin probe (SP-HP). (C) Monomeric non-palindromic hairpin probe (NP-HP). Lane numbers correspond to incubation time. Band intensities were quantified using ImageJ software to obtain gray values, which were used to estimate the relative integrity of each probe over time. The initial intensity at 0 h was defined as 100%. Experimental conditions: MF-HP = SP-HP = NP-HP = 500 nM.

To systematically assess the nuclease resistance of our A-MF-HP nanoarchitecture, we conducted comparative stability studies using three different probe designs: the current MF-HP containing dual palindromic sequences, a single-palindromic hairpin probe (SP-HP), and a non-palindromic hairpin probe (NP-HP) serving as the control. This design allowed for direct evaluation of the contribution of palindromic motifs-both single and dual-to the overall structural stability of the probes under nuclease-rich conditions. All probes used in this study were unlabeled with exogenous moieties such as FAM to eliminate potential interference from external chemical modifications. Each probe was exposed to accelerated degradation conditions in 10% human serum at 37 °C, and aliquots were analyzed by gel electrophoresis at specific time intervals. As shown in Fig. 5A, the A-MF-HP nanoarchitecture demonstrated remarkable biostability, maintaining its structural integrity for over 12 h in serum, with no significant reduction in band intensity compared to the 0 h control. This stability can be attributed to the compact superstructure formed by palindromic self-assembly, which provides steric protection against nucleases. Of note, the observed interfere band in pure human serum and all other lanes is likely attributable to residual circulating nucleic acids or native protein-nucleic acid complexes that remain intact under native electrophoretic conditions. The SP-HP variant (Fig. 5B) exhibited intermediate stability, maintaining detectable signals for approximately 3 h due to its ability to form dimeric SP-HP, which offers some protection. In contrast, the NP-HP control (Fig. 5C) rapidly degraded within 1 h, highlighting the vulnerability of monomeric probes to serum nucleases. To quantitatively evaluate probe biostability, the band intensities were analyzed using ImageJ software. The gray values of each band at different incubation times were normalized to the 0 h control, and the relative integrity of the probes was calculated accordingly. The results showed that A-MF-HP maintained more than 90% of its initial intensity after 12 h in 10% serum, whereas SP-HP retained only about 54% after 3 h of incubation, and NP-HP dropped to less than 51% within 1 h, indicating rapid degradation. These quantitative data are consistent with the qualitative gel observations, confirming that the dual-palindromic self-assembled structure provides substantially enhanced nuclease resistance compared with non-assembled or partially assembled probes. In addition to enhanced nuclease resistance, the self-assembled A-MF-HP provides functional advantages over monomeric probes such as TaqMan. Whereas TaqMan requires external PCR amplification, A-MF-HP integrates multiple recognition sites and enables cascade amplification under isothermal conditions. This design also improves stability in serum, making the system more suitable for direct use in complex biological samples. Although the tetrameric structure is more complex, this trade-off yields superior sensitivity and robustness. These features highlight the unique potential of A-MF-HP for reliable miRNA detection in practical applications.

### 3.9 Analysis of miRNA-21 in blood samples

**Fig. 6:**
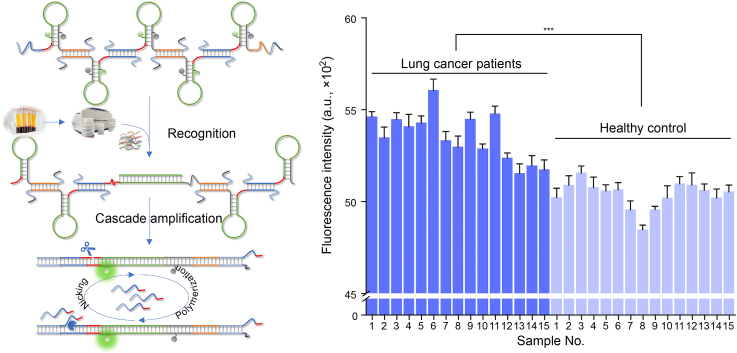
Brief illustration of the real sample detection procedure and fluorescence intensity comparison between blood samples from lung cancer patients (*n* = 15) and healthy controls (*n* = 15), following pre-treatment. Cancer patient samples exhibited higher fluorescence signals, indicating elevated microRNA-21 (miRNA-21) expression levels. Error bars represent the standard deviation of triplicate experiments. Statistical significance was assessed using Student's *t*-test (^∗∗∗^*P* < 0.001).

MiRNA-21 has been widely reported to be overexpressed in the blood of lung cancer patients, establishing it as a promising biomarker for early cancer diagnosis and prognosis [[41], [42], [43]]. To evaluate the clinical applicability of the A-MF-HP-based amplification system, we analyzed target miRNA from human blood samples based on above demonstrated features. The samples were categorized into two groups: lung cancer patients and healthy individuals. To minimize matrix interference from the complex biological background of whole blood, each sample underwent pre-treatment involving an annealing step and appropriate dilution prior to fluorescence analysis. As shown in Fig. 6, blood samples from lung cancer patients exhibited markedly higher fluorescence signals compared to those from healthy controls, indicating a significantly elevated level of circulating miRNA-21. Since the fluorescence intensity directly reflects the abundance of the target miRNA, these results corroborate previous clinical findings and confirm the diagnostic relevance of the A-MF-HP system. In parallel, the same set of samples was analyzed by RT-qPCR for comparison. As shown in Fig. S3A, lung cancer patient samples exhibited significantly higher signal intensities than healthy controls. The strong agreement between our assay and RT-qPCR confirms the robustness and diagnostic reliability of the A-MF-HP-based biosensor for circulating miRNA detection. Furthermore, ROC curve analysis (Fig. S3B) indicated acceptable diagnostic accuracy of this work (AUC = 0.9956) compared with the traditional RT-qPCR method (AUC = 0.9778), demonstrating excellent diagnostic performance in distinguishing patients from controls. The ability to clearly distinguish between patient and control groups highlights the potential of this method for reliable miRNA quantification in real samples. It is notable that, given the very limited presence of pre-miRNAs, cell-free DNA, and protein interference in human blood, future improvements may include a small RNA extraction step to remove pre-miRNAs and protein interference, and/or DNase pretreatment to eliminate potential DNA interference, thereby ensuring that the signal originates exclusively from mature miRNAs and further enhancing specificity in clinical applications.

## 4 Conclusions

In conclusion, we successfully developed a multifunctional nucleic acid probe and structurally self-assembled system (A-MF-HP) that simultaneously addresses the challenges of system complexity and limited biostability inherent in conventional nucleic acid detection methods. By integrating dual palindromic domains into a facile designed MF-HP, we enabled autonomous assembly into higher-order nanostructures that not only support efficient, enzyme-assisted cascade amplification but also enhance biostability with nuclease resistance. The A-MF-HP platform demonstrated excellent analytical performance, including femtomolar-level sensitivity, broad dynamic range, and high specificity toward miRNA-21, with the capacity to discriminate closely related sequences and mismatched variants. We acknowledge a trade-off between structural integration and operational simplicity: while the self-assembly and multifunctional features substantially enhance stability and signal fidelity, they also entail a more intricate probe design and optimization process than some streamlined homogeneous assays of comparable sensitivity, particularly those relying on single-component architectures. Nevertheless, this strategy offers a conceptually complementary approach that broadens the analytical repertoire for achieving sensitive and biostable nucleic acid detection, particularly in contexts where structural robustness and integrated functionality provide distinct practical advantages. Importantly, the probe retained its structural integrity under complex biological conditions, exhibiting prolonged biostability without compromising signal fidelity. Its successful application in clinical blood samples further highlights the potential of this platform for early cancer diagnosis and miRNA-based disease monitoring. Overall, this work opens a new path for future nucleic acid diagnostics by integrating rational structural design with functional versatility, resulting in a more robust, user-friendly, and adaptable probe technology.

## CRediT authorship contribution statement

**Baoqiang Chen:** Writing – original draft, Validation, Software, Investigation, Formal analysis. **Yiping Fan:** Software, Investigation, Formal analysis. **Qi Wang:** Validation, Software, Formal analysis. **Jianguo Xu:** Writing – review & editing, Validation, Supervision, Project administration, Methodology, Investigation, Conceptualization. **Yi Wang:** Resources, Formal analysis. **Bingyong Lin:** Formal analysis. **Lee Jia:** Writing – review & editing, Supervision, Project administration, Methodology, Formal analysis. **Longhua Guo:** Writing – review & editing, Project administration, Funding acquisition.

## Generative AI declaration

Generative AI tools were used solely for language editing, and all scientific content and conclusions are the responsibility of the authors.

## Declaration of competing interest

The authors declare that they have no known competing financial interests or personal relationships that could have appeared to influence the work reported in this paper.
