# Supplementary material for: Self-assembled multifunctional hairpin probe for ultrasensitive and mismatch-selective miRNA detection
Source: J Pharm Anal. 2026 Jan 22;16(7):101561. doi: 10.1016/j.jpha.2026.101561 (PMC13380003; doi:10.1016/j.jpha.2026.101561)
Supplement: Multimedia component 1 [file mmc1.docx]

**Supplementary materials for:**

**Self-assembled multifunctional hairpin probe for ultrasensitive and mismatch-selective miRNA detection**

**
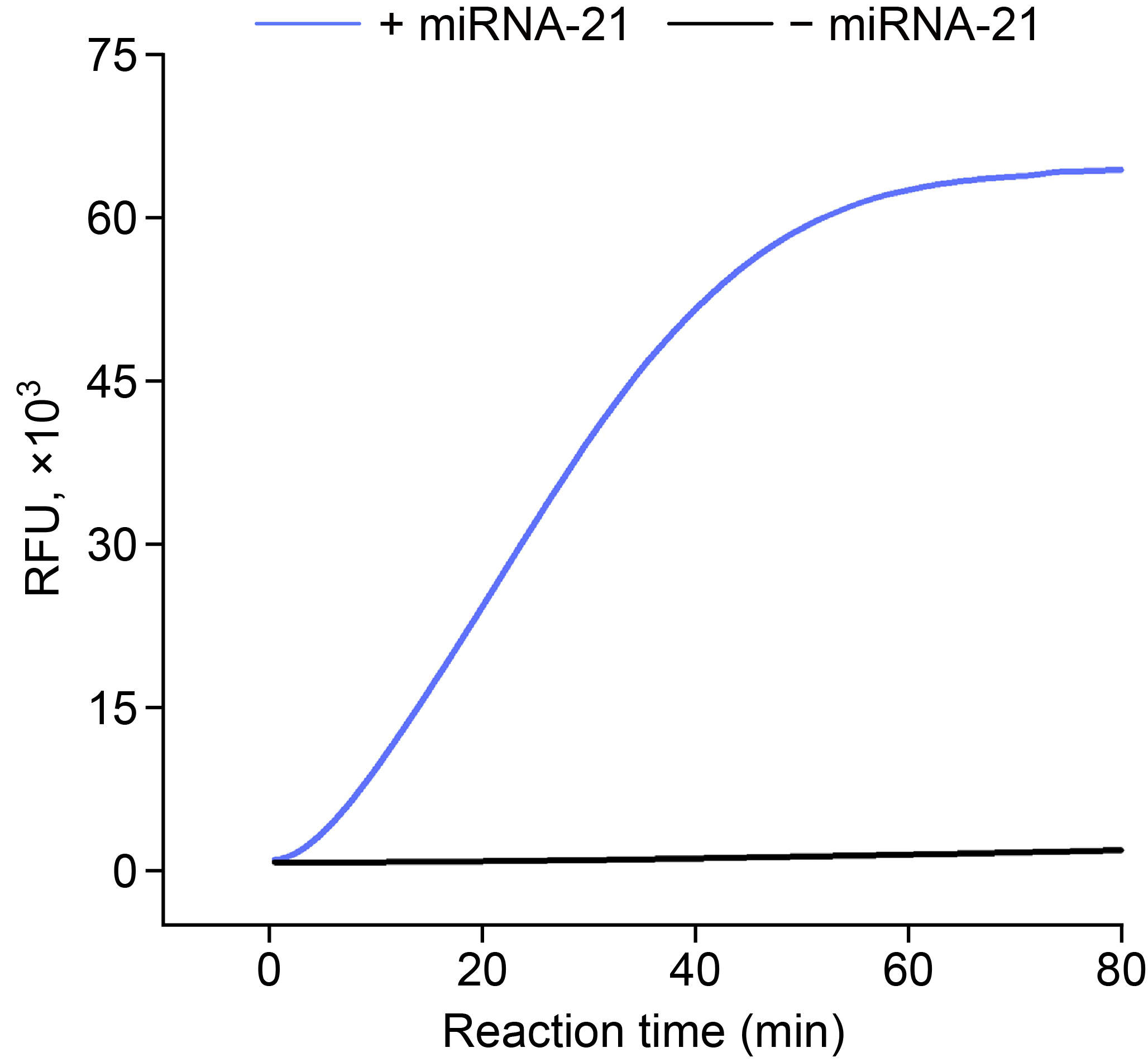
**

Fig. S1. Real-time fluorescence monitoring of the A-MF-HP system with and without miRNA-21. Kinetic fluorescence curves were recorded in the presence of 10 nM miRNA-21 (blue) and in the absence of target (black), showing rapid signal growth only when target was present.

**Reaction kinetics study**

To further investigate the dynamic behavior of the A-MF-HP system, real-time fluorescence measurements were conducted. As shown in Fig. S1, the presence of 10 nM miRNA-21 induced a rapid and continuous increase in fluorescence over 60 min, whereas negligible changes were observed in the absence of target. These results confirm that the amplification process is strictly target-dependent. The time-course data directly demonstrate the dynamic amplification mechanism and provide experimental validation of the cascade system’s reaction kinetics.

**
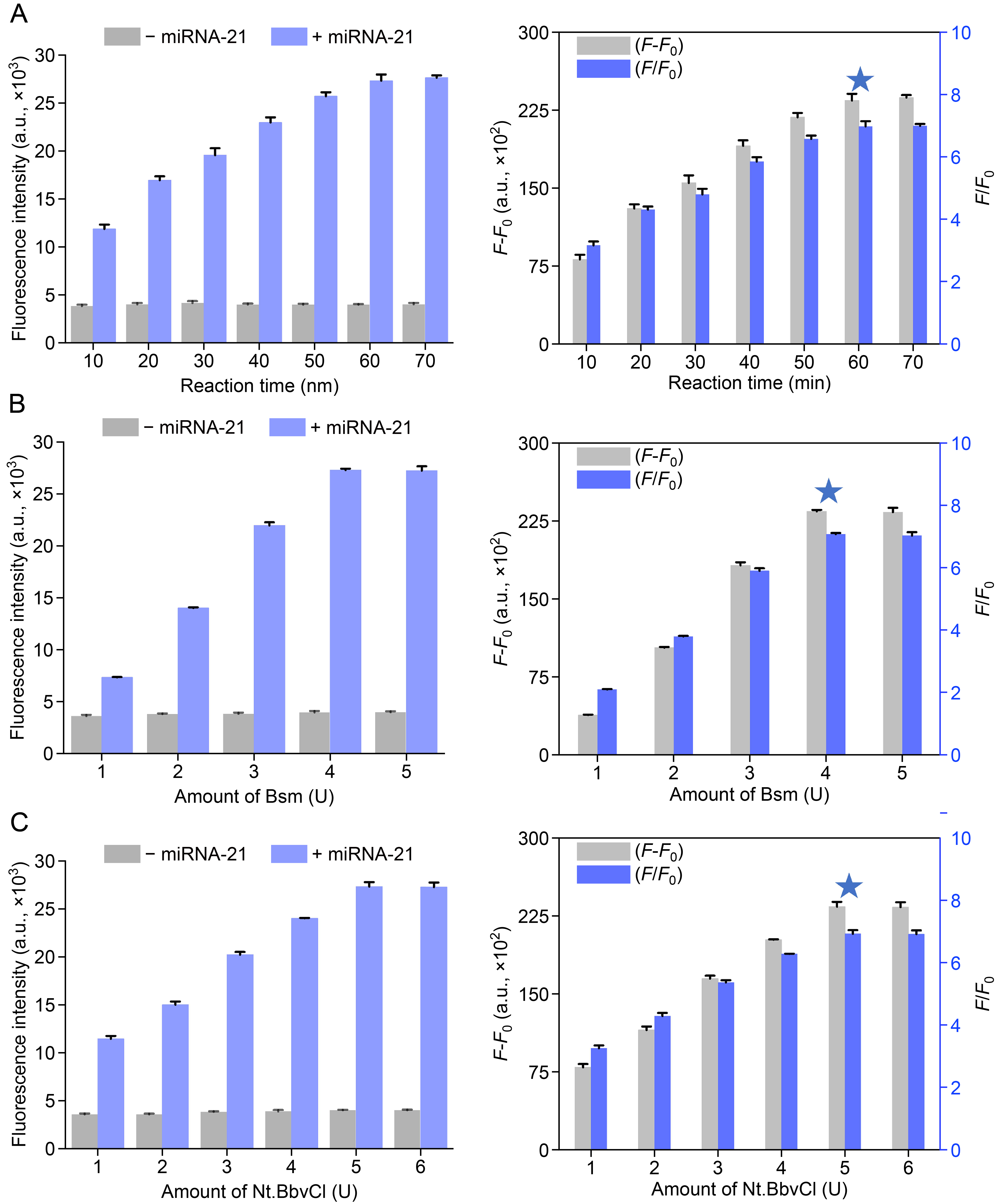
**

Fig. S2. Optimization of assay performance based on (A) enzymatic reaction time, (B) Bsm DNA polymerase concentration, and (C) Nt.BbvCI endonuclease concentration. Experimental conditions: (A) [miRNA-21] = 10 nM, [Bsm] = 20 U/mL, [Nt.BbvCI] = 25 U/mL, [dNTPs] = 125 μM, [MF-HP] = 20 nM; (B) [miRNA-21] = 10 nM, [Nt.BbvCI] = 25 U/mL, [dNTPs] = 125 μM, [MF-HP] = 20 nM, T = 60 min; (C) [miRNA-21] = 10 nM, [Bsm] = 20 U/mL, [dNTPs] = 125 μM, [MF-HP] = 20 nM, T = 60 min. Error bars represent the standard deviation from three independent experiments.

**Optimization of Experimental Conditions**

To optimize the assay performance, we evaluated critical experimental parameters, including reaction time and enzyme concentrations, which are essential for enzyme-based amplification. The concentration of MF-HP was maintained at 20 nM. Initially, we assessed the impact of reaction time on peak fluorescence intensity. As shown in the left panel of Fig. S2A, increasing the reaction time from 10 min to 60 min led to a continuous rise in target fluorescence, with no further increase observed after 60 min. The background signal remained unchanged across different time points. The net signal gain (F-F_0_) and signal-to-noise ratio (F/F_0_) were correspondingly calculated in the right panel of Fig. S2A. Based on these results, we set the reaction time to 1 h. Next, we investigated the effect of Bsm DNA polymerase concentration on fluorescence intensity (left panel of Fig. S2B). Initially, increasing the Bsm concentration enhanced the target signal, but eventually, the signal plateaued as MF-HP was consumed. The background signal remained unaffected by changes in Bsm concentration. Therefore, the optimal concentration of Bsm was determined to be 4 U according to the maximal signal increase in right panel of Fig. S2B. Finally, we optimized the concentration of Nt.BbvCI endonuclease (Fig. S2C). The trend observed was similar to that of Bsm, with an optimal Nt.BbvCI concentration of 5 U. These findings provide a foundation for subsequent experiments, ensuring maximal sensitivity and efficiency in the assay system.

**
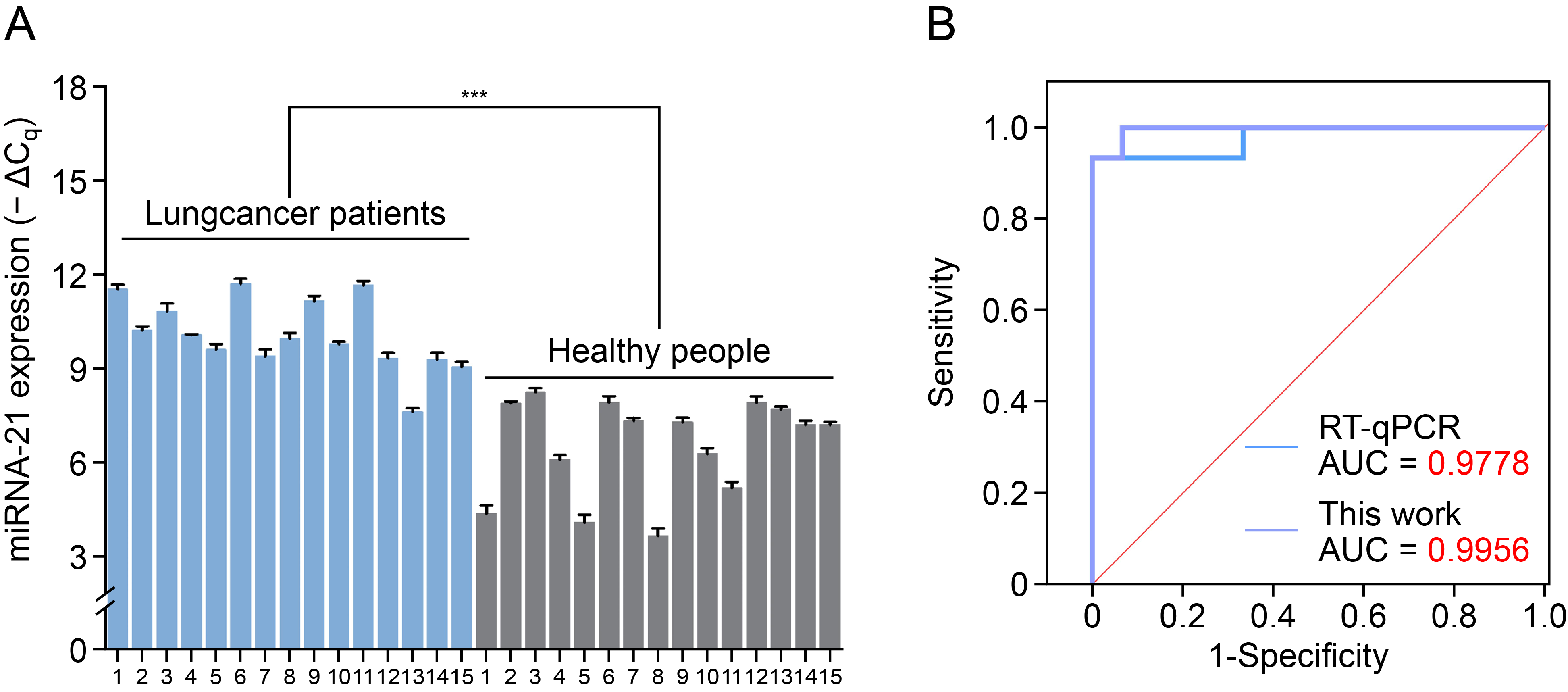
**

Fig. S3. (A) RT-qPCR analysis of miRNA-21 expression in serum samples from lung cancer patients and healthy controls, expressed as relative fold change using the -ΔCq method. (B) Receiver operating characteristic (ROC) curve analysis of the assay performance.

**Validation of Clinical Performance by RT-qPCR and ROC Analysis**

To further validate the clinical performance of the A-MF-HP assay, the same set of serum samples was analyzed in parallel by RT-qPCR. As shown in Fig. S3A, RT-qPCR confirmed significantly elevated expression levels of circulating miRNA-21 in lung cancer patients relative to healthy controls, consistent with the fluorescence assay results. In addition, ROC curve analysis was performed to evaluate diagnostic accuracy. As shown in Fig. S3B, the ROC curve analysis indicated acceptable diagnostic accuracy of this work (AUC = 0.9956) compared with the traditional RT-qPCR method (AUC = 0.9778), highlighting the A-MF-HP platform with excellent performance in differentiating patient samples from controls.

**Table S1**. Oligonucleotides used in the current study

| **Items** | **Sequences (5’ to 3’ order)** |
| --- | --- |
| MF-HP | TTGCTTCAGAGTCGACTC***GCTGAGG***T(FAM)CAACATCAGTCTGATAAGCTAGTT(BHQ1)GACCGTACGCGTACTTGCTTCATTTTT |
| SP-NP | TTGCTTCATTTTTTTTTT***GCTGAGG***TCAACATCAGTCTGATAAGCTAGTTGACCGTACGCGTACTTGCTTCATTTTT |
| NP-HP | TTGCTTCATTTTTTTTTT***GCTGAGG***TCAACATCAGTCTGATAAGCTAGTTGACCTTTTTTTTTTTTGCTTCATTTTT |
| ssDNA product | TCAGCGAGTCGACTCTGAAGCAA |
| miRNA-21 | UAGCUUAUCAGACUGAUGUUGA |
| MT1 | UAGCUUAUCAcACUGAUGUUGA |
| MT2 | UAGgUUAUCAGACUGAUcUUGA |
| MT3 | UAGgUUAUCAcACUGAUcUUGA |
| MT4 | UAGgUUtUCAGACUcAUcUUGA |
| IT1 | UAGCUUAUCA**C**GACUGAUGUUGA |
| DT1 | UAGCUUAUCAACUGAUGUUGA |
| miRNA-155 | UUAAUGCUAAUCGUGAUGGGGU |
| miRNA-141 | UAACACUGUCUGGUAAAGAUGG |
| let-7d | AGAGGUAGUAGGUUGCAUAGUU |
| miRNA-10b | UACCCUGUAGAACCGAAUUUGUG |
| miRNA-200b | UAAUACUGCCUGGUAAUGAUGA |
| RT miR-21 stem-loop | GTCGTATCCAGTGCAGGGTCCGAGGTATTCGCACTGGATACGACTCAACATCAGTCTGATAAGCTA |
| miR-21 forward primer | GCCGCTAGCTTATCAGACTGATGT |
| miR-21 reverse primer | GTGCAGGGTCCGAGGT |
| U6 RNA forward primer | CTCGCTTCGGCAGCACA |
| U6 RNA reverse primer | AACGCTTCACGAATTTGCGT |

Note: The bold and italicized bases in MF-HP indicate half of the Nt.BbvCI recognition site. The blue and orange bases represent palindromic sequences with reverse-complementary capabilities. The two shaded regions share identical sequences, with an additional five thymine (T) bases appended at the 3' end to prevent nonspecific amplification. In the SP-HP variant, the blue palindromic sequences of MF-HP are replaced with poly-T sequences. In the NP-HP variant, both the blue and red palindromic regions are substituted with poly-T sequences. Aside from these modifications, SP-HP and NP-HP retain the same design as MF-HP. The ssDNA product generated during the reaction shares an identical sequence. MT1, MT2, MT3, and MT4 represent mismatched miRNA targets, with mismatched bases shown in lowercase. IT1 contains one additional inserted base (in bold) compared to the miRNA-21 target, while DT1 has one base deleted. miRNA-155, miRNA-141, let-7d, miRNA-10b, and miRNA-200b are non-target control sequences. The stem-loop RT primer converts mature miRNA-21 into cDNA with high specificity. The miRNA-21 forward and reverse primers amplify this cDNA during qPCR, while U6 primers amplify U6 RNA as an internal control for normalization.

**Table S2**. Comparison of assay performances among various isothermal amplification methods for nucleic acid detection.

| **Detection Method** | **Target Molecule** | **LOD** | **Linear range** | **Detection time** | **Ref.** |
| --- | --- | --- | --- | --- | --- |
| Exponential amplification (EXPAR) reaction-triggered three-dimensional bipedal DNA walkers | miRNA-21 | 5.2 fM | 10 fM to 5 nM | 70 min | [1] |
| An intermolecular and intramolecular priming co-directed synergistic multi-strand displacement amplification | miRNA-21 | 1.18 fM | 10 fM to 25 nM | 120 min | [2] |
| The exponential amplification reaction | let-7a | 5.84 fM | 10 fM to 10 nM | 40 min | [3] |
| Dual ferrocene-labeled DNA hairpin based on Mg^2+^-dependent DNAzyme-cleavage cycling | miRNA-21 | 1.8 fM | 10 fM to 100 pM | 150 min | [4] |
| CRISPR/Cas12a-Assisted Ligation-Initiated Loop-Mediated Isothermal Amplification | let-7a | 0.1 fM | 0.1 fM to 100 fM | 50 min | [5] |
| Chemoreceptive biosensor based on layered graphene oxide/graphene composite | miRNA-21 | 14.6 pM | 10 pM to 100 nM | 420 min | [6] |
| Target-triggered hybridization chain reaction | miRNA-21 | 1.92 fM | 5 fM to 10 pM | 150 min | [7] |
| Bidirectional palindromic assembly of a multifunctional hairpin probe facilitating robust cascade signal amplification | miRNA-21 | 1.0 fM | 1 fM to 10 nM | 60 min | This study |

**References**

[1] L. Yang, J. Fang, J. Li, et al., An integrated fluorescence biosensor for microRNA detection based on exponential amplification reaction-triggered three-dimensional bipedal DNA walkers, Anal. Chim. Acta 1143 (2021) 157-165.

[2] J. Xia, Z. Liu, S. Gao, et al., Intermolecular and intramolecular priming co-directed synergistic multi-strand displacement amplification empowers ultrasensitive determination of microRNAs, Anal. Chem. 94 (2022) 16132-16141.

[3] H. Guo, J. Chen, Y. Feng, et al., A simple and robust exponential amplification reaction (EXPAR)-based hairpin template (exp-Hairpin) for highly specific, sensitive, and universal microRNA detection, Anal. Chem. 96 (2024) 2643-2650.

[4] X. Lin, J. Jiang, J. Wang, et al., Competitive host-guest recognition initiated by DNAzyme-cleavage cycling for novel ratiometric electrochemical assay of miRNA-21, Sens. Actuators B Chem. 333 (2021) 129556.

[5] M. Zhang, H. Wang, H. Wang, et al., CRISPR/Cas12a-Assisted Ligation-Initiated Loop-Mediated Isothermal Amplification (CAL-LAMP) for Highly Specific Detection of microRNAs, Anal. Chem. 93 (2021) 7942-7948.

[6] C.-H. Huang, T.-T. Huang, C.-H. Chiang, et al., A chemiresistive biosensor based on a layered graphene oxide/graphene composite for the sensitive and selective detection of circulating miRNA-21, Biosens. Bioelectron. 164 (2020) 112320.

[7] Y. Wang, H. Feng, K. Huang, et al., Target-triggered hybridization chain reaction for ultrasensitive dual-signal miRNA detection, Biosens. Bioelectron. 215 (2022) 114572.
